# Biventricular endomyocardial fibrosis with right ventricular hypertrabeculation and rapid progression to heart transplantation: imaging and pathologic correlation

**DOI:** 10.31744/einstein_journal/2026RC2390

**Published:** 2026-09-02

**Authors:** Sofia Alves Figueiredo Faustino, Ana Clara Tude Rodrigues, Ebe Christie de Oliveira, João de Azevedo, Fernando Hideki Sakamoto, Claudio Henrique Fischer, Sandrigo Mangini, Edgar Lira-Filho, Samira Saady Morhy, Ana Olga H Mocumbi, Marcelo Luiz Campos Vieira

**Affiliations:** 1 Hospital Israelita Albert Einstein São Paulo SP Brazil Hospital Israelita Albert Einstein, São Paulo, SP, Brazil.; 2 Universidade Eduardo Mondlane Maputo Moçambique Universidade Eduardo Mondlane, Maputo, Moçambique.

**Keywords:** Endomyocardial fibrosis, Noncompacted myocardium, Heart transplantation, Echocardiography, Athlete's heart

## Abstract

Endomyocardial fibrosis is a restrictive cardiomyopathy characterized by the deposition of fibrotic tissue in the endocardium, predominantly affecting the ventricular apices and atrioventricular valves. Although classically described in endemic regions and low socioeconomic settings, its pathophysiology remains incompletely understood. The coexistence of endomyocardial fibrosis with noncompacted myocardium has not been previously reported. Here, we describe a 57-year-old man, an amateur endurance cyclist, who presented with a two-year history of progressive heart failure symptoms, including fatigue, ascites, and lower limb edema. Transthoracic echocardiography revealed severe biatrial enlargement, grade III diastolic dysfunction, valvular regurgitation, and apical obliteration of both ventricles. Marked right ventricular trabeculation with deep intertrabecular recesses and turbulent flow was observed, raising suspicion of non-compacted myocardium. Despite optimized medical therapy, the patient experienced recurrent hospitalizations and progressed to advanced restrictive heart failure, requiring orthotopic heart transplantation. Pathological examination of the explanted heart confirmed chronic-stage endomyocardial fibrosis with extensive endocardial fibrosis and prominent right ventricular trabeculation. This case demonstrates a previously unreported association between endomyocardial fibrosis and right ventricular hypertrabeculation, expanding the phenotypic spectrum of endomyocardial fibrosis and emphasizing the importance of comprehensive imaging assessment in atypical presentations.

## INTRODUCTION

Endomyocardial fibrosis (EMF) is a cardiomyopathy characterized by the deposition of fibrotic tissue in the endocardium, primarily involving the apical regions of the ventricles and the atrioventricular valves.^(1)^ It has been predominantly reported in Africa, Latin America, and South Asia, although its prevalence has declined in some of these regions. Its pathophysiology remains incompletely understood^(1)^ but may involve factors such as low socioeconomic status, malnutrition, genetic predisposition, and parasitic diseases.^(2,3)^ The coexistence of noncompacted myocardium (NCM) and EMF has not been previously documented. Here, we report a patient with EMF and a possible association with non-compacted myocardium, who experienced rapid progression to advanced heart failure requiring heart transplantation.

## CASE REPORT

A 57-year-old man, an amateur cyclist with a history of asthma, presented with fatigue on moderate exertion, lower limb edema, ascites, and orthopnea that had developed over two years. Initial evaluation at another institution included transthoracic echocardiography and cardiac magnetic resonance imaging, which demonstrated obliteration of the apical portions of both the left ventricle (LV) and the right ventricle (RV), with preserved biventricular ejection fraction. These findings were consistent with EMF. Heart failure (HF) therapy was initiated; however, the patient experienced recurrent hospitalizations for clinical decompensation. Coronary angiography revealed no significant coronary artery disease, and he was referred to our center because of refractory HF symptoms. On admission, the patient appeared emaciated, with massive ascites and marked lower limb edema. Laboratory testing showed mild eosinophilia and stage 3a chronic kidney disease, with an estimated creatinine clearance of 50mL/min/1.73m². Diagnostic paracentesis revealed exudative ascites with low inflammatory markers. Transthoracic echocardiography demonstrated severe biatrial enlargement, mild RV dilation with systolic dysfunction, preserved LV systolic function, and severe grade III diastolic dysfunction. Apical obliteration of both ventricles was evident, with increased echogenicity consistent with fibrotic tissue (Figure 1A). In the RV, fibrous tissue extended unusually into the outflow tract. A small pericardial effusion was also present (Figure 1B). Color Doppler imaging revealed marked trabeculation in the RV apical and free-wall regions, with turbulent flow within deep intertrabecular recesses, raising suspicion of non-compacted myocardium or persistent myocardial sinusoids (Figure 1B). No similar trabeculation was observed in the LV. Valvular assessment showed mild-to-moderate mitral regurgitation and severe tricuspid regurgitation. Global longitudinal strain analysis revealed markedly reduced strain values, particularly in the apical segments. Despite aggressive medical therapy, the patient experienced further clinical deterioration and was diagnosed with advanced restrictive heart failure (stage D). Inotropic support was required, and he was ultimately listed for orthotopic heart transplantation, which he underwent without perioperative complications. Gross pathological examination of the explanted heart revealed diffuse and marked thickening of the endocardium in both ventricles, with extensive fibroplasia and hyalinized collagen deposition. Fibrosis extended into the underlying myocardium, with fibrous septations reaching toward the subepicardial layers (Figure 2A). The RV showed prominent trabeculations with wide intertrabecular recesses (Figure 2B), particularly in the lateral free wall, whereas the LV exhibited no abnormal trabeculation. Valvular involvement with fibrotic changes was evident.

**Figure 1:**
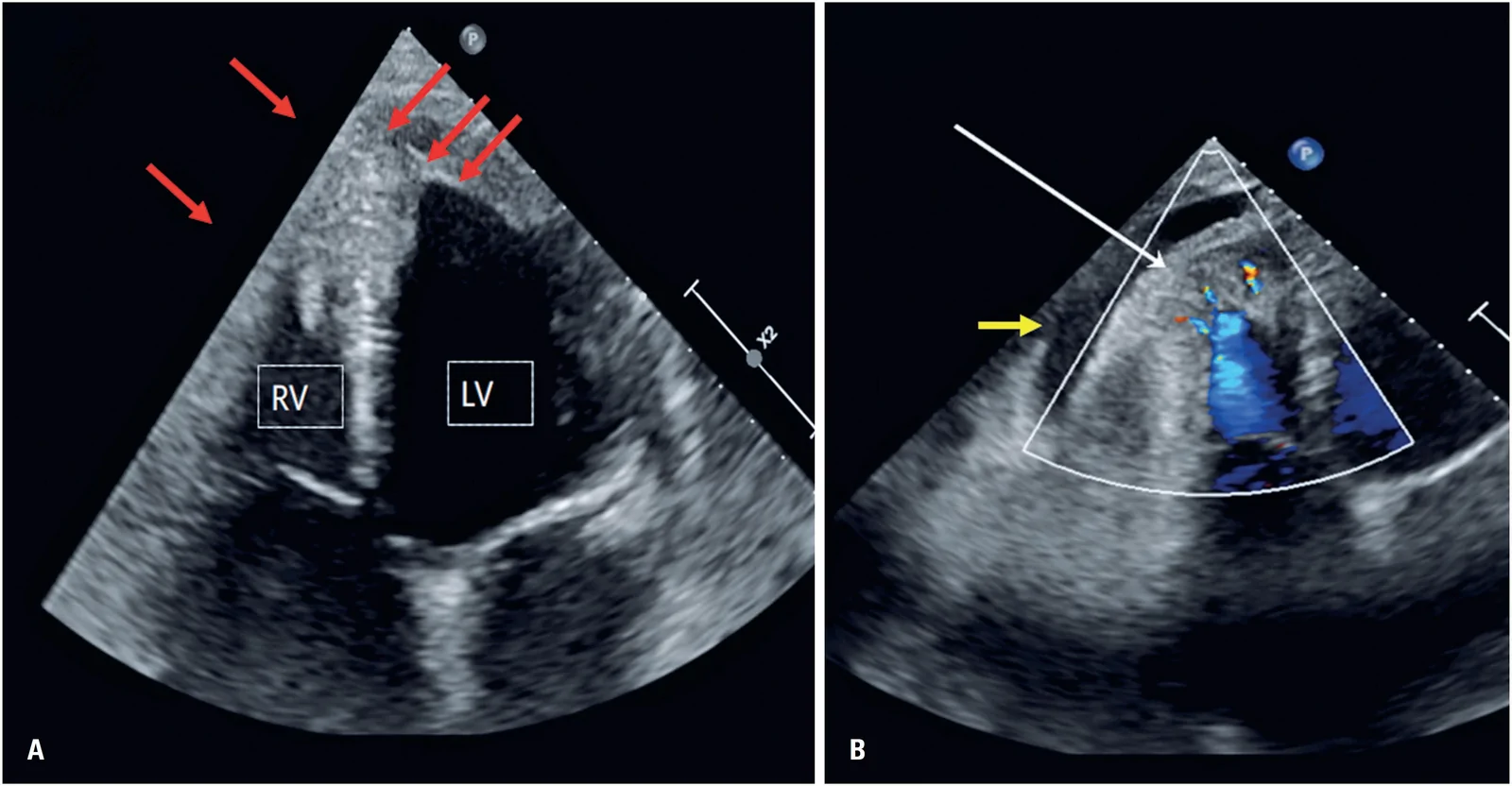
Echocardiogram (4-chamber view) showing apical filling of the ventricles (A) with fibrosis (red arrows), and (B) prominent trabeculations in the apical region of the right ventricle, with turbulent flow within the trabeculations (white arrow). A small pericardial effusion is seen (yellow arrow)

**Figure 2:**
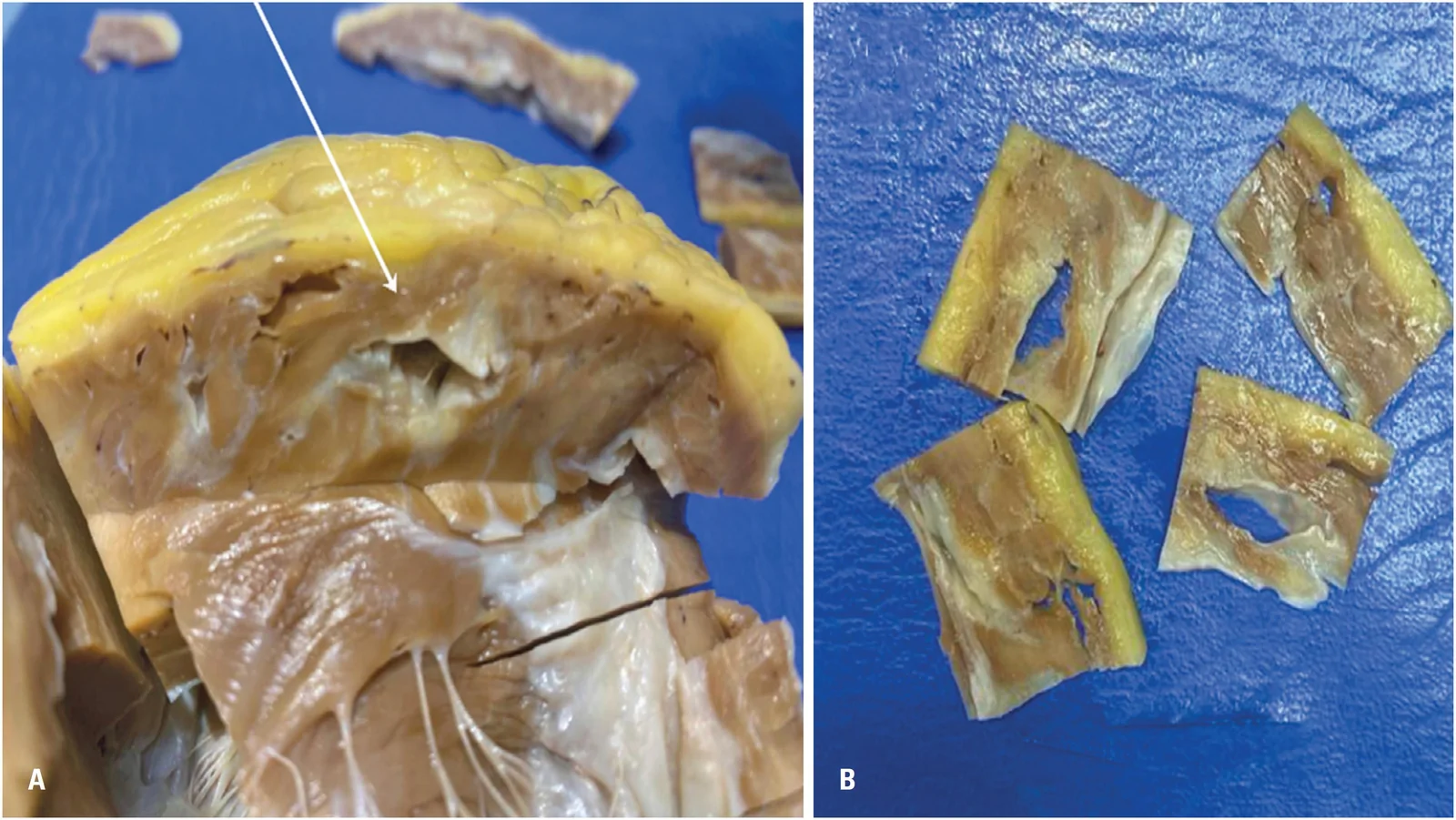
Macroscopic examination of the explanted heart showing diffuse thickening of the endocardium, with multiple empty spaces amidst fibrosis (white arrow), best observed in thin sections of the heart (B)

Histological analysis confirmed dense endocardial fibrosis composed predominantly of collagen, with sparse cellular connective tissue and no evidence of tissue eosinophilia. The findings were consistent with chronic-stage EMF. The patient remained clinically stable and asymptomatic one year after transplantation.

The study was approved by the Research Ethics Committee of the *Hospital Israelita Albert Einstein*, CAAE: 85130224.9.0000.0071; # 7.279.196.

## DISCUSSION

This case illustrates an unusual presentation of biventricular EMF associated with marked RV trabeculation suggestive of noncompacted myocardium. Endomyocardial fibrosis is classically described in patients from low socioeconomic backgrounds in endemic regions;^(1)^ however, our patient had a high socioeconomic status and no history of parasitic disease. Mild asthma was present, which may be relevant given the known association between eosinophilia and the early inflammatory phase of EMF. However, peripheral blood eosinophilia was only mild, and no tissue eosinophilia was observed on histological examination, consistent with advanced disease.

The chronic phase of EMF is characterized by restrictive physiology, preserved systolic function, severe diastolic dysfunction, biatrial enlargement, valvular regurgitation, reduced myocardial deformation, and pericardial effusion,^(3)^ all of which were present in this patient. Ascites is frequently exudative, as observed here, reflecting elevated systemic venous pressures and chronic inflammation. Although fibrosis typically spares the ventricular outflow tracts, our patient demonstrated significant RV outflow tract involvement, an uncommon finding.^(3)^

The most striking feature was the presence of excessive RV trabeculation with deep blood-filled recesses, observed both on echocardiography and on pathological examination (Figure 1). This appearance contrasts with the usual pattern of EMF, in which fibrotic infiltration leads to trabecular compaction and exclusion of the trabecular portion from ventricular circulation. Noncompacted myocardium is classically characterized by excessive trabeculation and deep intertrabecular recesses^(4)^ most commonly affecting the LV, whereas RV involvement is less frequently reported.^(5)^ Its pathogenesis remains controversial, and it is increasingly viewed as a morphological phenotype that may be associated with various cardiac or systemic conditions, including athletic remodeling.^(6,7)^ Given the patient's history as an amateur endurance athlete, RV hypertrabeculation could represent a pre-existing adaptive response, although prior imaging was unavailable to confirm this possibility. Alternatively, the trabecular pattern may represent an atypical response to EMF-related remodeling or an adaptive mechanism for preserving endocardial perfusion in the setting of extensive fibrosis. The absence of LV trabeculation and the coexistence of classic EMF pathology argue against primary diffuse noncompaction cardiomyopathy, supporting instead a possible localized or secondary phenomenon.

Treatment options for EMF are limited. Medical therapy addresses heart failure symptoms but does not halt disease progression. Surgical resection of fibrous tissue with valve repair or replacement remains the main therapeutic option for symptomatic patients^(8)^ although it carries significant perioperative risk and is often unavailable in endemic regions. Heart transplantation has emerged as a viable option for selected patients with advanced disease^(9,10)^ as demonstrated by this case.

## CONCLUSION

Endomyocardial fibrosis is a rare and severe restrictive cardiomyopathy that may progress rapidly to advanced heart failure. We report a unique case of biventricular endomyocardial fibrosis associated with prominent right ventricular hypertrabeculation suggestive of noncompacted myocardium, an association that has not been previously described. Heart transplantation resulted in an excellent clinical outcome. This case expands the phenotypic spectrum of endomyocardial fibrosis and raises questions regarding myocardial remodeling and trabeculation in advanced fibrotic cardiomyopathies.

## Data Availability

The underlying content is contained within the manuscript.
